# Foreign Correspondence

**Published:** 1847-01

**Authors:** 


### THE WESTERN JOURNAL.

New Series.—Vol. VII.—No. I.

LOUISVILLE, JANUARY 1, 1847.

### FOREIGN CORRESPONDENCE.

The following letter from our Foreign Correspondent ought to have reaehed us a fortnight before it came to hand, in which case it would have been appended to the one which appears in another part of this number. His descriptions of the eminent men of Paris will be read with interest. We have already received from him a letter of a more professional character, which will appear in our next number.

Paris, Oct. 22d, 1846.

Gentlemen: In Paris there is at this time a young physician from Massachusetts, diligently engaged in the study of *Veterinary Surgery*, with reference to delivering lectures on that subject when he returns home. He has the appearance of being an intelligent, gentlemanly, studious young man, but I fear that he will not be rewarded for his labor. We are hardly yet sufficiently advanced in civilization to appreciate acquirements in that truly interesting branch of science. A "horse doctor" in the United States is one thing; a veterinary surgeon in France is quite another thing. I do not pretend to affirm that our horse doctors are more ignorant and illiterate than the common tribe of empirics; but the Paris veterinary surgeon has a rank among scientific men, and his profession is esteemed a dignified and honorable one; nor can any one who regards the true position of the horse in the scale of being, and considers our dependence upon that noble animal wish to see it otherwise. The distinguished London surgeon, Mr. Liston, during a visit which he paid not long since to Paris, was more interested in the veterinary hospital and school than in the hospitals and schools for our own species. In a future communication I shall have something to say of this institution.

I have just had the pleasure of seeing the renowned astronomer, Le Verrier, who, as I mentioned in a former letter, is only thirty-one years of age. He is about the middle stature, with fair complexion, light hair, and a mild, benevolent expression of face bordering on tameness. He would strike four out of every five Americans as some country schoolmaster. You have already seen, no doubt, that a Mr. Adams, of Cambridge, England, disputes with Le Verrier the honor of the discovery which has so suddenly rendered him famous. The English astronomers are resolved that the new planet shall not be named after the French discoverer. To the world at large the naming of a star is a matter of small moment, and the animated controversy to which it has given rise, however exciting to astronomers, is dull enough to most readers. One of my friends here, who feels an intense concern as to who shall bear the palm, predicts that England is bound eventually to add this gem also to the diadem with which Newton and Herschel have encircled her brow.

M. Arago, so eminent as a mathematician, I have also seen at the Academy of Sciences. He is advancing in years, and his head is quite gray; his height is six feet two inches, and his appearance very fine. His features, which are strongly marked, are rather indicative of energy and force of character, than of that excelling intellect which has placed him foremost among the members of the French Academy. His eyebrows are absolutely immense, and hang like thunder clouds over his large mild eyes.

The name of Regnault is familiar to all who have watched the recent progress of physical science. He is, I presume, the youngest member of the Academy, and certainly is the most ordinary looking.

He is a small young man, of fair complexion and light hair, with nothing noticeable in his face, person or manner. The only thing with which you would be struck on learning that he was the acute and profound philosopher whose researches have already given him a name in all lands where science is cultivated, is the real "soap-lock" cut and length of his hair. When I looked at him and thought of his great attainments, I was forcibly reminded of the lines:

> " 'Tis not the front or visage of a man
> That marks his merit."

He read a long and elaborate paper to the Academy the evening I saw him, on the compressibility of elastic fluids, which was much complimented by the members, but upon me the greater portion of it was lost, owing to the rapidity with which he hurried through his essay. At a late meeting of the Academy of Sciences I saw the chemists Thenard and Chevreul, and had the satisfaction of hearing a paper read by the latter on *chemical reaction*. Chevreul is about five feet eleven inches high, fifty or fifty-five years of age, with long, uncombed gray hair, small head inclining to be pointed, prominent thin nose, and black, deeply sunken eyes, which are never at rest. In manner, while reading, he is earnest and rapid, sawing up and down from his table to the back of his chair, and gives one the idea of a restless, eccentric man. Thenard is large, between fifty and sixty years old, of florid complexion, large head, curly hair, small nose, wide face, black eyes, prominent cheek bones, and a stern expression of countenance. He sits with his chin upon his breast, apparently absorbed in thought, but when he rises to speak his manner is pleasing and impressive.

One of the greatest men, physically, with whom you will meet in Paris, and also a great man, intellectually, is Rayer, one of the physicians to La Charité, whose name is known to the profession in our country. He is an immense man, forty-five or fifty years old; two hundred and twenty pounds would hardly pull him down. He has a fine face, with black, excessively long hair, and straight as an Indian's, black eyes, and a mouth which, though small, is expressive of pride and determination.

Dr. Desmarres has a small private class, of which I am a member, to which he lectures on diseases of the eye, three months, and for this course receives from each pupil no more than ten francs. He has in the press and will shortly publish a treatise on those diseases, of which I will avail myself of the earliest opportunity to give you some account, for, from my knowledge of the author, I am persuaded that it will be a work of merit. He is still a young man, of good size, and rather good appearance, notwithstanding a pug nose. His face is exceedingly animated and expressive while lecturing, and I regard him as one of the best teachers with whom I have met. With the patients before his class, his remarks, and the black-board, upon which he nearly every day makes drawings explanatory of his subjects, his course is very clear, interesting and valuable. He has five *cliniques* a week, of two hours' each; the number of patients daily present is from thirty-five to forty; his method of instruction is to assign, almost every day, a patient to each student who diagnosticates and prescribes; after which Desmarres corrects, if the prescription or diagnosis requires it, and concludes by making some remarks on the nature and treatment of the disease. After the student has made sufficient progress, he is entrusted with the knife in cases demanding an operation. To-day a case was submitted to me for diagnosis and prescription. In the first I was successful, but my treatment did not please the doctor, and so he ordered a different course. But "doctors," you know, "*will* differ." I cannot conceive of a better school for studying affections of the eye than the cliniques of Dr. Desmarres.

